# The Future of Orthopædic Surgery

**Published:** 1920-12-18

**Authors:** 


### THE FUTURE OF ORTHOPÆDIC SURGERY.

The term " orthopædic " is a bad one, as it does not cover the group of cases included in these days, which, according to Sir Robert Jones, K.B.E., F.R.C.S., should consist of :

(*a*) Fractures—recent, malunited, and ununited.

(*b*) Congenital and acquired deformities of the extremities.

(*c*) Paralyses of the extremities.

(*d*) Diseases, derangements, and disabilities of the joints, including the spine, wry neck, and those conditions included under the general term " bone-setting."

This definition covers a very large area of surgery, and is in sharp contrast to the orthopædic surgery of a decade ago, when lateral curvature, flat feet, knock knees, and a few congenital deformities mainly sufficed to represent the speciality.

The census of 1921 will give us definite knowledge of the numbers of crippled people in this country. They must be very large, and it is unfortunate that at the present time we have neither sufficient hospital accommodation to ward them nor enough trained orthopædic surgeons to treat them. Thanks to the war, many general surgeons gained a competent knowledge of orthopædics, but many more indeed are needed. The days when a general surgeon was expected to operate on anything from the enucleation of tonsils to the worst forms of flat feet are long since past, and he should no longer be asked to undertake them. In opening the annual meeting of the British Orthopædic Association a few weeks ago Sir Robert Jones gave his ideas as to the constitution of a model orthopædic department attached to a general hospital.

### A Model Orthopædic Department.

He defined the scope of orthopædic surgery so as to avoid its encroachment upon other branches of surgery, and claimed that no orthopædic department should be confined to the treatment of out-patients, but should be a complete unit, with beds, commensurate to out-patient demands.

He suggests that the staff should consist of

(*a*) An orthopædic surgeon, who should be in charge of the department and see to its efficient conduct. He should be responsible for all cases admitted to his department, and have a seat on the Medical Board, holding equal rank with the general surgeon; furthermore, his practice out of the hospital should be confined to orthopædic surgery.

(*b*) An orthopædic registrar, who should be a Fellow of the College or studying for his final examination, and should be selected as a man desirous of practising orthopædic surgery.

(*c*) A house surgeon, who should be appointed for at least six months.

(*d*) Dressers and clinical assistants.

*Nursing.*—It is important that the matron should grant security of tenure to the sisters in charge of the orthopædic out-patient department and wards, because it takes time to train nurses in the special work involved.

*Beds.*—In a hospital of 300 surgical beds we should aim at obtaining no fewer than forty for the orthopædic service, and these beds should be segregated and not mixed with other types of cases. The out-patient department should contain a complete physio-therapy department, a plaster room (preferably in close proximity to the operating theatre), a plaster-modelling room, a photographic room, and a workshop for the manufacture of splints and appliances. In order that complete records may be kept, *a secretary should be attached to each unit.*

We would also suggest that a staff of out-patient visitors might well be attached to such a department, so that supervision of diet and suggestions as to suitable personal hygiene be given to the relations of the patient.

### Open-air Country Hospitals.

The great majority of cripples are children, and the futility of treating such as in-patients in the vitiated atmosphere of city hospital wards is now generally recognised.

Over twenty years ago Sir Robert Jones, with the help of Miss Hunt, started the first really open-air hospital in the world, where children live in the open air by day and night all the year round. This was followed by the large country hospital at Heswall, and later at Ruislip and Harrow. In none of these hospitals is there any possibility of closing the wards, and through sunshine and storm, snow and sleet, one side of the shed is always absent.

At the present time the governors of the Royal National Orthopædic Hospital have adopted this principle, and are now appealing for funds to build and fully equip a country branch, where most of the operations will be performed.

In conclusion, we would mention that Sir George Newman, the chief medical officer of the Board of Education, has recently called attention to the existing state of affairs in his annual report, and after the next census has revealed the actual numbers of cripples, we shall confidently expect the Ministry of Health to formulate a national scheme for the treatment of those patients at present untreated, or needing more efficient care.

